# Molecular characterization and morphological description of cryptic haemoproteids in the laughingthrushes (Leiothrichidae) in the western and eastern Himalaya, India

**DOI:** 10.12688/wellcomeopenres.14675.1

**Published:** 2018-08-01

**Authors:** Farah Ishtiaq, Megha Rao, Vaidas Palinauskas

**Affiliations:** 1 Centre for Ecological Sciences, Indian Institute of Science, Bangalore, Karnataka, 560012, India; 2 Institute of Ecology, Nature Research Centre, Akademijos 2, Vilnius, 2100, LT-08412, Lithuania

**Keywords:** cryptic species, Haemoproteus, India, Laughingthrushes

## Abstract

**Background:** Laughingthrushes (family: Leiothrichidae) consists of diverse and widespread species found in the Indian subcontinent but there is a lack of information on their avian haemosporidians.

**Methods**: We sampled 231 laughingthrushes of 8 species in the western and eastern Himalaya in India. Using parasite morphology and cytochrome *b* sequences we describe 2 new *Haemoproteus* species harbored in 3 species of laughingthrushes and report a case of cryptic speciation.

**Results**: First *Haemoproteus* lineage TROERY01 (GenBank: KY623720) found in *Trochalopteron erythrocephalum* (27.47%) and *Trochalopteron variegatum *(2.9%) in mid to high altitude tropical forests in the western and eastern Himalaya, was described as *Haemoproteus (Parahaemoproteus) leiothrichus *n. sp. (Haemosporida: Haemoproteidae). Second *Haemoproteus* lineage TROERY02 (GenBank: KY623721) described as *Haemoproteus (Parahaemoproteus) homoleiothrichus *n. sp. (Haemosporida: Haemoproteidae) was found in *T. erythrocephalum *(2.19%) and *Trochalopteron lineatum* (3.84%), albeit in low intensity, only in the western Himalaya. Both *H. homoleiothrichus *n. sp. and *H. leiothrichus* n. sp. showed no significant difference in morphological features in blood stages. A genetic divergence of 4.4% along with distinct phylogenetic position indicates that these 2 lineages represent cryptic species. Previously, *T. erythrocephalum* has been described as an additional host for a morphologically described *Haemoproteus timalus *in the oriental region. Our described species have several morphological features that are absent in *H. timalus.* These are, the presence of dumbbell-like shaped mature gametocytes, ‘arm’ like extensions of gametocytes and lateral displacement of nuclei of infected erythrocytes. Illustrations of blood stages of the new species are given, and phylogenetic analysis with morphologically described *Haemoproteus *species identifies parasites closely related to the 2 described parasites.

**Conclusions**: The lineages described here have been recorded only in the laughingthrushes so far. These are the first parasites to be described with *T. erythrocephalum *as a type host from the western and eastern Himalaya in India.

## Introduction

Avian haemosporidians are cosmopolitan, vector-borne, and intracellular parasites, first discovered back in 1880 by V. Ya. Danilewsky ( Valkiūnas, 2005). These parasites are placed in 3 genera: *Plasmodium*, *Haemoproteus* or *Leucocytozoon* ( Atkinson & van Riper, 1991). Currently, the genus *Haemoproteus* consists of over 130 species based on morphological descriptions of erythrocytic stages ( Valkiūnas *et al.*, 2008). Since the advent of molecular techniques (e.g., Feldman *et al.*, 1995), avian haemosporidians have been used as a popular model system to understand epidemiology and host-parasite co-evolution. Recent molecular studies have revealed marked diversity in avian haemosporidians and over 2,000 unique lineages have been characterized based on mitochondrial cytochrome *b* (cyt *b*) gene ( Bensch *et al.*, 2009). Determining the diversity of these vector-mediated parasites is hindered by large knowledge gaps in complete life-cycles, and the lack of combined molecular and morphological descriptions ( Clark *et al.*, 2014; Outlaw & Ricklefs, 2014). Linking between haemosporidian DNA lineages and their morphospecies has remained a major challenge which is fundamental to our understanding the cryptic speciation and evolution of host-parasite relationships. Recent studies on avian haemosporidians have recorded the presence of cryptic parasite species ( Nilsson *et al.*, 2016; Palinauskas *et al.*, 2015; Sehgal *et al.*, 2006). In particular, Nilsson *et al.* (2016), has recently drawn conclusion using molecular techniques that five lineages nested within *Haemoproteus majoris*, represent five different biological species and are morphologically cryptic.

In the absence of experimental manipulations to characterize cryptic species, it is often impossible to understand the complete stages of life cycle and to determine competent vector groups which can provide crucial information in identification of transmission areas. Nevertheless, for a species to be defined as cryptic, the morphology with respect to developmental and sexual stages of the parasite species, genetic distinctness, phylogenies and host range provide convincing evidence ( Nilsson *et al.*, 2016; Palinauskas *et al.*, 2015; Perkins, 2000; Sehgal *et al.*, 2006).

The Indian subcontinent has an exceptional diversity of avifauna with around 1400 species ( Rasmussen & Anderton, 2005). Ideally, such high diversity should reflect upon the diversity of avian haemosporidians in the tropics which provide a great opportunity to exploit a broad range of avian hosts. However, studies on avian haemosporidians which can support this prediction are lacking in the Indian subcontinent. Bennett *et al.* (1991) and Nandi & Bennett (1997) were the main studies solely based on the morphology and estimates of avian haemosporidian prevalence across a large scale survey in the Indian sub-continent. More recently, Ishtiaq *et al.* (2007) used both microscopy and molecular techniques to screen avian haemosporidians in 43 species of birds from India.

During an ongoing study on the diversity and distribution of avian haemosporidians across eastern and western Himalaya in India, blood samples from eight species of laughingthrushes (Leiothrichidae) were collected, in the mid-elevation forests. Here, we describe 2 prevalent *Haemoproteus* species in laughingthrushes using morphological analysis of blood stages and mitochondrial cyt *b* gene sequences. These are the first parasites to be described infecting *Trochalopteron erythrocephalum* as a type host from the western and eastern Himalaya in India.

## Methods

### Ethical approval

The field experiments comply with the current laws of the India where the study was performed. We thank Uttarakhand Forest Department and Arunachal Pradesh Forest Department for permission for the collection of avian blood samples.

### Bird sampling

**Figure 1.**
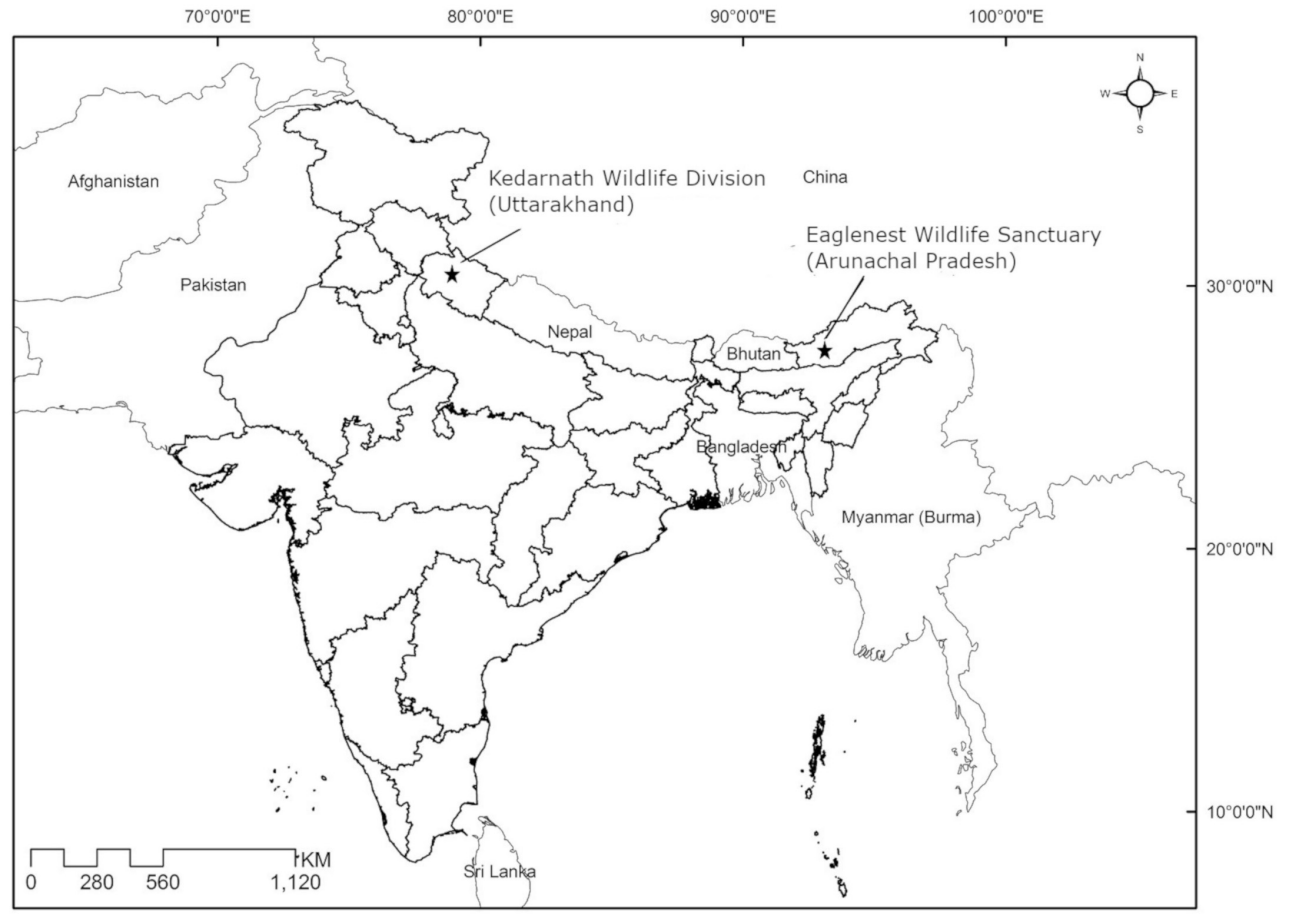
Study sites for laughingthrush sampling in India.

Bird sampling was conducted between April-May in Kedarnath Wildlife Division (30.457042 N, 79.274714 E; 1,500–3,000 meters above sea level [masl]), Uttarakhand (western Himalaya) from 2014–2016 and during April 2014 in Eaglenest Wildlife Sanctuary (27.1 N, 92.4 E; 2,000 masl), a global biodiversity hotspot, Arunachal Pradesh in the Eastern Himalaya in India ( Figure 1). Our samplings coincide with the peak breeding season of birds. In total, 231 samples from laughingthrushes from 8 species were used in this study (Dataset 1 ( Ishtiaq, 2018)). Birds were sampled using mist-nets. Each bird caught was identified using Rasmussen & Anderton (2005), ringed to avoid resampling and released at the site soon after processing. About 30 µl of blood (never exceeding 1% of individual’s body weight) was drawn from the brachial vein. The samples were collected in SET buffer (20–40 μL in 500 μL buffer 0.15M NaCl, 0.05M Tris, 0.001M EDTA, pH 8.0) or on FTA cards (catalogue no. Z719730, Whatman, GE Healthcare, Buckinghamshire, England) and were later transferred to the lab and stored at -20° C for subsequent genetic analysis.

Simultaneously, we made 2 or 3 thin blood smears from each bird. All blood smears were fan or air dried within 5–10 sec after their preparation and fixed with absolute methanol on field as described in Valkiūnas (2005). Blood smears were Giemsa-stained (catalogue no. 51811-82-6, Fisher Scientific, Mumbai, India) along with phosphate buffer within a month of preparation in the field camp following Valkiūnas (2005) protocols.

### Microscopy

In total, 103 out of 231 samples with blood smears were microscopically examined. A Leica DM1000 compound microscope equipped with Leica EC3 camera (Wetzlar, Germany) and imaging software imageJ 1.x ( Schneider *et al.*, 2012) was used to examine slides, prepare illustrations and take measurements. The smears were first examined for 10–15 min at low magnification (×500), and then at least 100 fields were studied at high magnification (×1,000). Intensity of infection was estimated as a percentage by actual counting of the number of parasites per 1,000 red blood cells or per 10,000 red blood cells if infections were light, i.e., <0.1%, as recommended by Godfrey *et al.* (1987). To determine the possible presence of simultaneous infections with other haemosporidian parasites in the type material of new species, the entire blood films from hapantotype and parahapantotype series were examined at low magnification.

### Morphological analysis

The morphometric features studied ( Table 1) are those defined by Valkiūnas (2005). Morphology of new species was compared with voucher specimens of *Haemoproteus timalus* (accession numbers 4060 and 4062) from the blood of the *Garrulax mitratus*, in the collection of the International Reference Centre for Avian Haematozoa (IRCAH) at the Queensland Museum, Australia.

**Table 1.** Morphometry of host cells and mature gametocytes of *Haemoproteus leiothrichus* sp. nov. and *Haemoproteus homoleiothrichus* sp. nov from *Trochalopteron erythrocephalum* and comparison with *Haemoproteus timalus* ( Bennett *et al.*, 1991). All measurements are in micrometers with means followed by the standard deviation in parenthesis. Nuclear displacement ratio (NDR) follows Bennett & Campbell (1972).

	*H. leiothrichus* n. sp.	*H. homoleiotrichus* n. sp.	*Haemoproteus timalus*
**Uninfected erythrocyte**	**( *n*=20)**	**( *n*=20)**	**( *n*=85)**
Length	11.57 (0.74)	10.94 (0.74)	11.60 (0.6)
Width	6.18 (0.46)	6.63 (0.35)	6.60 (0.5)
Length of nucleus	4.46 (0.32)	4.39 (0.48)	5.00 (0.3)
Width of nucleus	2.93 (0.31)	2.94 (0.51)	2.40 (0.2)
**Erythrocyte parasitized by** **macrogametocyte**	**( *n*=10)**	**( *n*=10)**	**( *n*=85)**
Length	11.93 (0.75)	12 (0.84)	12.70 (0.7)
Width	5.53 (0.44)	6.79 (0.72)	6.40 (0.6)
Length of nucleus	3.63 (0.38)	4.23 (0.31)	4.80 (0.4)
Width of nucleus	2.57 (0.18)	3.25 (0.4)	2.20 (0.3)
**Erythrocyte parasitized by** **microgametocyte**	**( *n*=10)**	**( *n*=8)**	**( *n*=40)**
Length	12.86 (1.21)	11.18 (0.79)	12.80 (0.7)
Width	6.56 (0.36)	6.79 (0.49)	6.40 (0.5)
Length of nucleus	4.37 (0.3)	4.68 (0.39)	4.90 (0.4)
Width of nucleus	3.23 (0.26)	3.08 (0.39)	2.30 (0.3)
**Macrogametocyte**	**( *n*=10)**	**( *n*=10)**	**( *n*=85)**
Length	10.27 (0.64)	10.58 (0.94)	12.50 (1.0)
Width	3.79 (1.07)	4.9 (0.61)	2.20 (0.5)
Length of nucleus	2.24 (0.35)	1.99 (0.36)	2.60 (0.4)
Width of nucleus	1.57 (0.26)	1.81 (0.42)	2.00 (0.4)
NDR	0.53 (0.33)	0.316 (0.20)	0.80 (0.2)
No of pigment granules	8.02 (1.58)	9.4 (2.0)	11.30 (2.2)
**Microgametocyte**	**( *n*=10)**	**( *n*=10)**	**( *n*=40)**
Length	12.49 (0.82)	10.55 (1.04)	13.40 (1.7)
Width	4.67 (0.58)	4.98 (0.96)	2.50 (0.5)
Length of nucleus	6.11 (0.83)	6.02 (0.77)	6.90 (0.8)
Width of nucleus	1.86 (0.22)	1.69 (0.28)	2.30 (0.4)
NDR	0.60 (0.33)	0.37 (0.12)	0.80 (0.10)
No of pigment granules	8.60 (1.6)	9.8 (2.1)	10.90 (2.2)

### DNA extraction, polymerase chain reaction (PCR), and sequencing

DNA was extracted from whole blood samples using the Phenol Chloroform Isoamyl extraction method ( Sambrook *et al.*, 1989). A restriction enzyme-based diagnostic PCR assay using 213F/ 372R primers (Sigma-Aldrich 13485;160 bp, mitochondrial ribosomal RNA (rRNA), cyt *b*; Beadell & Fleischer, 2005) that amplifies 3 genera of haemosporidians as well as detects mixed infections was used to screen the samples. For samples that screened positive for the diagnostic PCR, we amplified the cyt *b* gene fragments of *Plasmodium* and *Haemoproteus* using 3760F/4292RW2 (Thermofisher Scientific 4304970, 533 bp; Beadell *et al.*, 2004) or with nested PCR protocol using HAEM F/ HAEM R2 (Thermofisher Scientific 4304970, 479bp; Hellgren *et al.*, 2004). Positive and negative controls were used with each PCR reaction. The positive controls were from infected birds, as determined by microscopic examination of blood films or PCR, and the negative control was nuclease free water in place of DNA template.

PCR products were purified using Exosap-IT (Affymetrix, USB, Santa Clara, California) following manufacturer’s instructions and were sequenced using the Big Dye version 3.1 (Applied Biosystems, Foster City, California) in both directions. Sequences were assembled, aligned and edited using SEQUENCHER version 4.6 (GeneCodes, Ann Arbor, Michigan). Co-infections were identified by double nucleotide peaks on sequence electropherograms.

We then compared the obtained lineages to confirm the parasite genus with reference parasite sequences deposited in GenBank and in the MalAvi database ( Bensch *et al.*, 2009). All unique sequences are deposited in GenBank (accession numbers: KY623720 and KY623721).

### Phylogenetic analysis

**Figure 2.**
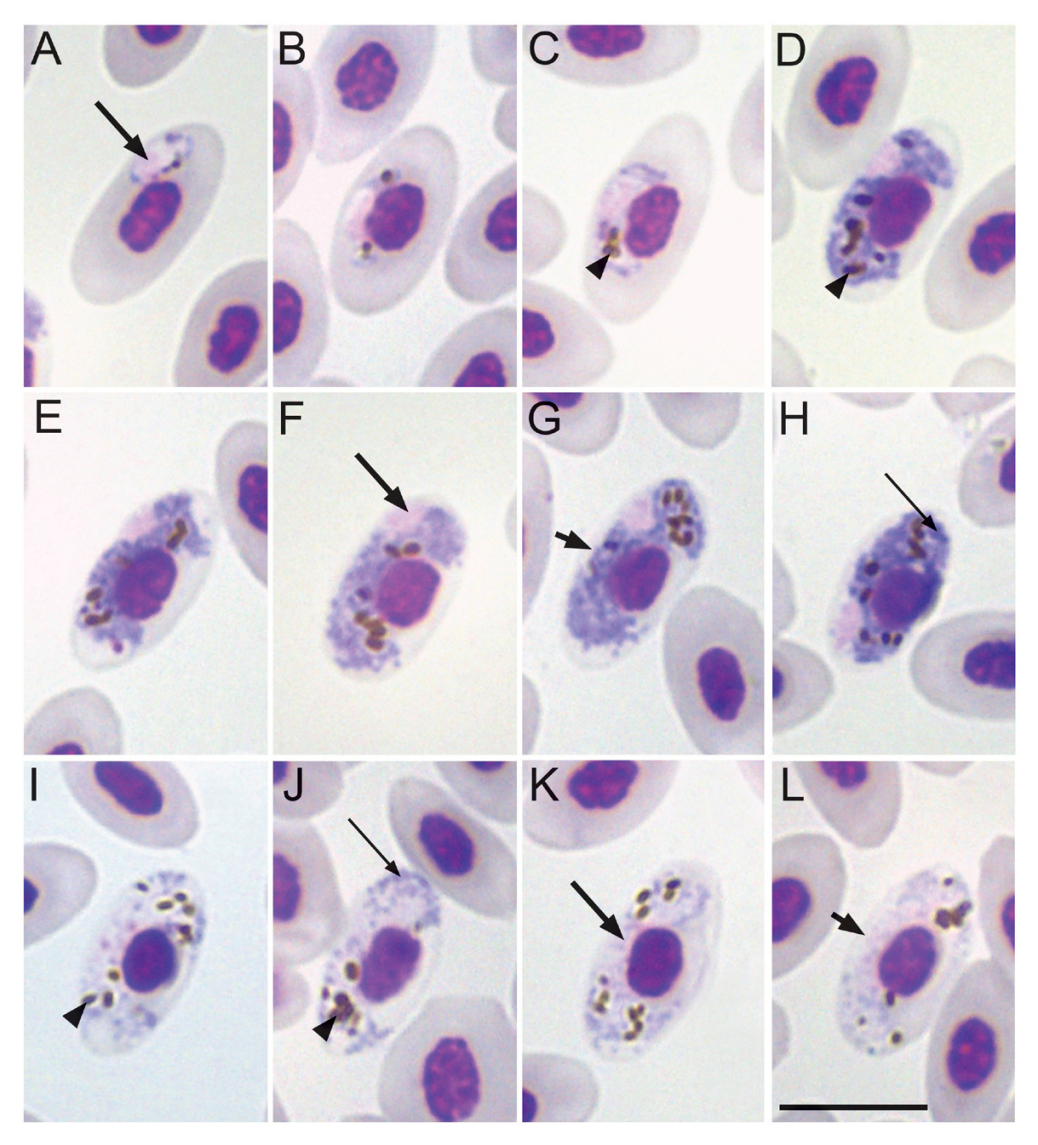
Gametocytes of *Haemoproteus leiothrichus* n. sp. ( **A**– **C**) Growing gametocyte, ( **D**– **H**) mature macrogametocyte and ( **I**– **L**) mature microgametocytes. Scale bar = 10 µm. Long arrow points to the nucleus of the parasite; short arrow - indicates unfilled spaces between gametocytes and envelope of infected erythrocyte; arrow head shows pigment granules; long narrow arrow shows volutin granules.

To place sequenced lineages in a phylogenetic context, we combined 30 cyt *b* sequences of the parasites identified and described as *Haemoproteus* here with 40 morphologically described avian *Haemoproteus* spp. from the MalAvi database ( Bensch *et al.*, 2009). All individual sequences were grouped into a 479 bp long consensus with *Leucocytozoon majoris* (GenBank accession number: AY393804) used as an outgroup ( Figure 2). We constructed the maximum likelihood tree using Bayesian phylogenetics as implemented in BEAST version 1.4.3 ( Drummond & Rambaut, 2007) and the most appropriate substitution model (GTR+G) according to the Akaike Information Criterion was implemented MEGA version 5.2 ( Tamura *et al.*, 2011). We present a Maximum Clade Credibility (MCC) tree using a relaxed molecular clock approach ( Drummond & Rambaut, 2007). Rates of substitution were drawn from a lognormal distribution and Yule prior was used for branching rates. We conducted 2 runs of 20 million generations, each with sampling conducted every 1,000 generations. *Tracer* version 1.6 was used to assess convergence, whether 2 chains were mixing and whether the estimated sample size (ESS) for each parameter was of sufficient size (ESS>200) to obtain robust parameter estimates. Four million generations were discarded as burn-in from each run, leaving a posterior distribution of 32,000 trees. Pair-wise genetic distance was calculated using Kimura 2-parameter evolution model in MEGA version 5.2 ( Tamura *et al.*, 2011).

## Results

Based on molecular methods, 105/231 (45.45%) samples were positive for avian haemosporidian infections. Of the positives, 38 samples showed mixed infections with *Plasmodium* and *Leucocytozoon* using restriction enzyme-based digest assay (Dataset 1 ( Ishtiaq, 2018)). Using microscopy technique, 49/103 showed infections with haemosporidians which suggest 52.42% were sub-microscopic infections. Of the positives, 12 showed mixed infections in microscopy.

Of the 105 positives, using nested PCR protocol we retrieved 30 *Haemoproteus* sequences from three species of laughinghtrushes: the most prevalent lineage described here as *Haemoproteus leiothrichus* n. sp. was found in 27.47% (n = 25/91) of *T. erythrocephalum* and 2.9% (n = 1/34) in *T. variegatum* (Dataset 1 ( Ishtiaq, 2018)). Second lineage described here as *H. homoleiothrichus* n. sp. was found in 2.19% (n = 2/91) of *T. erythrocephalum* and 3.84% (n = 2/52) of *T. lineatum* (Dataset 1 ( Ishtiaq, 2018)). We describe morphology of two new parasite species from single infections in host species.

### Description

***Haemoproteus (Parahaemoproteus) leiothrichus n. sp.*** *Diagnosis of young gametocytes* ( Figure 2A–C): The earliest stages of the parasite are not restricted to one position in the erythrocyte and were seen in polar, median and sub-polar positions ( Figure 2A). Growing gametocytes are mostly seen in the median position with respect to the erythrocyte ( Figure 2B–C). Young gametocytes adhere to the envelope of the erythrocyte as well as the nucleus of the erythrocyte ( Figure 2C). Irrespective of the position of the young gametocyte, the parasite as it grows occupies the median position growing towards both the nucleus and the envelope of the infected erythrocyte.

*Macrogametocytes* ( Figure 2D–H): Gametocytes grow along the nuclei of the infected erythrocytes, laterally displacing the nuclei of the infected erythrocytes ( Figure 2F, H). Mature macrogametocytes are usually closely associated with both the nuclei and the envelope of the erythrocyte. However, at times, there is also an unoccupied space observed between the parasite and the envelope of the erythrocyte even in mature macrogametocytes, causing a ‘dip’ and giving a dumbbell-like appearance ( Figure 2G). Mature macrogametocytes enclose the nucleus of the infected erythrocyte with their ends without encircling it completely ( Figure 2G, H). Macrogametocytes of this parasite have the tendency to almost fill one of the poles of the erythrocyte first while the other pole still remains unfilled and more often do not fill both poles of the erythrocyte ( Figure 2D, E, G, H). The gametocytes also extend so called an 'arm' ( Valkiūnas, 2005) across the erythrocyte, thus touching the opposite envelope of the erythrocyte ( Figure 2D, G). The outline of the gametocyte varies from amoeboid (>70%) to even. Nuclear displacement is observed in fully grown gametocytes where the nucleus is pushed laterally ( Figure 2H). Parasite nucleus is compact and roundish, sometimes variable in shape, located in a sub-median to sub-polar position. The nucleus of the parasite more often adheres to the envelope of the erythrocyte, but in some cases it was also seen adhering to the nucleus of the erythrocyte. Pigment granules are round to oval in shape and scattered ( Figure 2H), or sometimes slightly aggregated ( Figure 2G) in the cytoplasm of the parasite. The size of the pigment granules is most often medium whereas small pigment granules have also been observed. The average number of pigment granules is 8–9 per gametocyte. Vacuoles were not observed in the parasite. Volutin granules are present ( Figure 2H).

*Microgametocytes* ( Figure 2I–L): General configuration as for macrogametocytes, with main haemosporidian sexually dimorphic characters. The nucleus of the microgametocyte is diffused and median in position. Mature microgametocytes more often fill the poles of the infected erythrocyte ( Figure 2I–L). Volutin granules are present ( Figure 2I–K).

### Taxonomic summary

*Type host: Trochalopteron erythrocephalum* (Passeriformes, Leiothrichidae).

*DNA sequences:* Mitochondrial cyt *b* lineage 479 bp, GenBank accession number KY623720, TROERY01 lineage code.

*Additional hosts: Trochalopteron variegatum* (Variegated laughingthrush).

*Etymology:* The parasite is named as *Haemoproteus leiothrichus*, as it is found in *Trochalopteron erythrocephalum* which belongs to the family Leiothrichidae formerly included in Timaliidae.

*Site of infection:* Mature erythrocytes, no other data.

*Type locality:* Uttarakhand (30°45.704'N, 79°27.471'E, 1,500–3,000 masl) and Arunachal Pradesh (27°6.0'N, 92°24.0'E, 2,000 masl), India.

*Type specimen:* Hapantotype ( *Trochalopteron erythrocephalum,* Shokharakh (3,000 m) Kedarnath Wildlife Division, Uttarakhand State, India), intensity of parasitemia is 1% in *Trochalopteron erythrocephalum* deposited with voucher number ESM 201 in Ecological Sciences- Microorganism (ESM) collection at Centre for Ecological Sciences, Indian Institute of Science, Bangalore, India.

**Figure 3.**
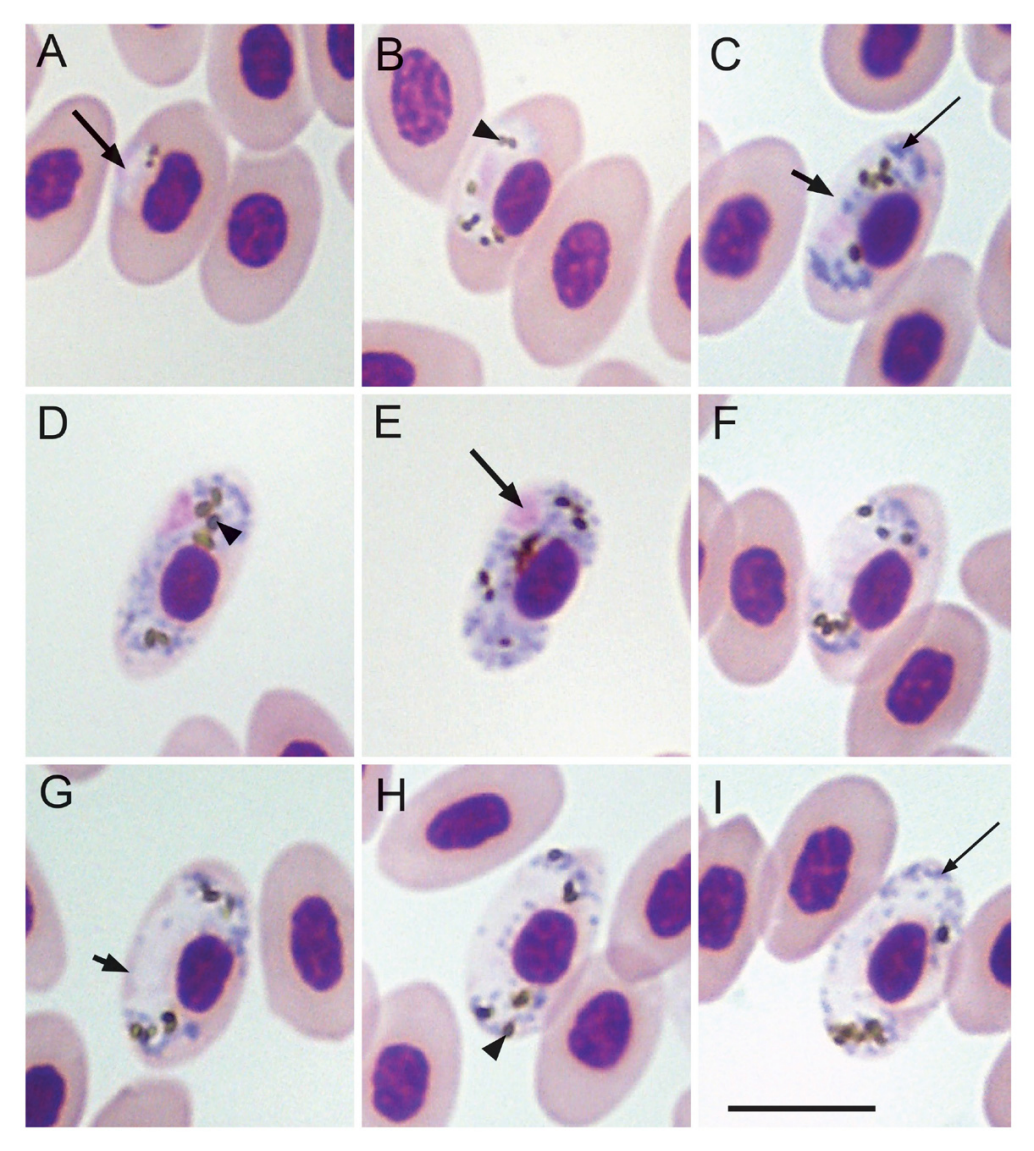
Gametocytes of *Haemoproteus homoleiothrichus* n. sp. ( **A**– **C**, **F**) Growing gametocyte, ( **D**, **E**) mature macrogametocyte and ( **G**– **I**) mature microgametocytes. Scale bar = 10 µm. Long arrow points to the nucleus of the parasite; short arrow - indicates unfilled spaces between gametocytes and envelope of infected erythrocyte; arrow head shows pigment granules; long narrow arrow shows volutin granules.

***Haemoproteus (Parahaemoproteus) homoleiothrichus n. sp.*** *Diagnosis of young gametocytes* ( Figure 3A, B): The earliest forms were seen in polar, median and sub-polar positions and do not seem to be restricted to single position in the erythrocyte ( Figure 3A). Later stage growing gametocytes have an even outline and are more often seen in the median position with respect to the erythrocyte ( Figure 3B–C). Young gametocytes adhere to the envelope of the erythrocyte as well as the nucleus of the erythrocyte ( Figure 3A). The parasite as it grows, occupies the median position adhering to both the nucleus and the envelope of the infected erythrocyte.

*Macrogametocytes* ( Figure 3C–E): Gametocytes grow along the nuclei of the infected erythrocytes, laterally displacing the nuclei of the infected erythrocytes. Macrogametocytes are closely associated with both the nuclei and the envelope of the erythrocytes ( Figure 3D, E). Mature macrogametocytes enclose the nucleus of the infected erythrocyte with their ends without encircling it completely ( Figure 3D, E). The outline of the gametocyte is more even (>70%) than amoeboid, even though amoeboid arms are sometimes seen. Dumbbell shaped forms are present ( Figure 3C, D). As the gametocyte grows, the nucleus is pushed laterally and thus the parasite occupies all the area of the cytoplasm of the infected erythrocyte ( Figure 3E). Some of the macrogametocytes of this parasite have the tendency to almost fill one of the poles of the erythrocyte first while the other pole still remains unfilled ( Figure 3D). Nuclear displacement is prominent and observed in growing as well as fully grown gametocytes where the nucleus is pushed laterally ( Figure 3C–E). Parasite nucleus is compact and roundish in shape, located in a sub-median to sub-polar position. The nucleus of the parasite adheres to the envelope of the erythrocyte. Pigment granules are round to oval in shape and scattered throughout the cytoplasm of the parasite ( Figure 3D). The size of the pigment granule ranges from small to medium. The average number of pigment granules is 8–10 per gametocyte. The cytoplasm contains volutin granules, vacuoles were absent.

*Microgametocytes* ( Figure 3F–I): General configuration as for macrogametocytes, with main haemosporidian sexually dimorphic characters. The nucleus of the microgametocyte is diffused and median in position. Mature microgametocytes 'almost' fill the poles of the infected erythrocyte and often push the nucleus of the infected erythrocyte laterally ( Figure 3F–I).

### Taxonomic summary

*Type host: Trochalopteron erythrocephalum* (Passeriformes, Leiothrichidae).

*DNA sequences:* Mitochondrial cyt *b* lineage 479 bp, GenBank accession number KY623721, TROERY02 lineage code.

*Additional hosts: Trochalopteron lineatum* (Streaked laughingthrush) *.*

*Etymology:* The parasite is named as *Haemoproteus homoleiothrichus*, as it is found in *Trochalopteron erythrocephalum* which belong to the family Leiothrichidae and is morphologically identical to *H. leiothrichus*.

*Site of infection:* Mature erythrocytes, no other data.

*Type locality:* Uttarakhand (30°45.704' N, 79°27.471' E, 1,500–3,000 masl)

*Type specimen:* Hapantotype ( *Trochalopteron erythrocephalum,* Mandal (1,500–1,800 m), Kedarnath Wildlife Division, Uttarakhand State, India), intensity of parasitemia is 1% in *Trochalopteron erythrocephalum* deposited with voucher number ESM 202 in Ecological Sciences- Microorganism (ESM) collection at Centre for Ecological Sciences, Indian Institute of Science, Bangalore, India.

### Molecular description

**Figure 4.**
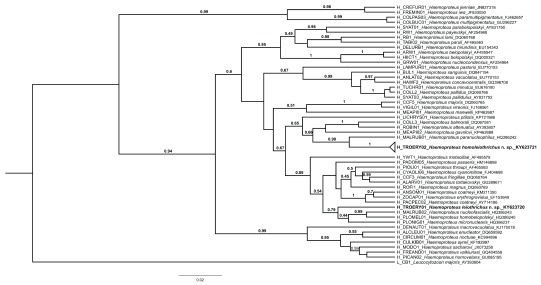
Bayesian phylogeny of 40 *Haemoproteus* spp. lineages described morphologically together with *Haemoproteus leiothrichus* sp. nov. (TROERY01) and *Haemoproteus homoleiothrichus* sp. nov. (TROERY02), the lineages recorded in the *Trochalopteron erythrocephalum* based on mitochondrial cytochrome *b* gene. *Leucocytozoon majoris* (GenBank Accession no: AY393804) has been used as an outgroup. Bootstrap values with greater than 60% support have been mentioned on the branches. Names of new species are given in bold.

Partial mitochondrial cyt *b* sequences of *H. leiothrichus* n. sp. and *H. homoleiothrichus* n. sp. isolated from the infected type host, *Trochalopteron erythrocephalum* (n = 27). The new lineage, TROERY01, represents the described new species – *Haemoproteus leiothrichus* n. sp., which falls within a well-defined monophyletic clade restricted to laughingthrushes ( Figure 4). *H. leiothrichus* n. sp. differs from other morphologically described *Haemoproteus* lineages by 1.7 – 10.6%. The other lineage, TROERY02 is *H. homoleiothrichus* n. sp. found in laughingthrushes (n = 4) falls in a separate clade and differs genetically by 2.1–12.7% from other morphologically described *Haemoproteus* lineages and shows a genetic distance of 4.4% from TROERY01 ( Figure 2; Table S1, Supplementary File 1).

### Remarks

*Haemoproteus leiothrichus* n. sp. and *H. homoleiothrichus* n. sp. share key morphological features; however, their difference in overall prevalence, distribution, phylogeny and a genetic divergence of 4.4% attributes to 2 separate parasite species. Both *Haemoproteus leiothrichus* n. sp. and *H. homoleiothrichus* n. sp. have gametocytes growing along the nuclei of the infected erythrocyte ( Figure 2F–H, Figure 3D, E). Dumbbell-like appearance is observed in the macro- and microgametocytes of both species ( Figure 2G, H, L, Figure 3C, D, G). A morphological feature observed in *H. leiothrichus* n. sp. and *H. homoleiothrichus* n. sp. is the tendency in some of the gametocytes to leave one of the poles unfilled even as lateral nuclear displacement is clearly observed ( Figure 2G, H, Figure 3D). The characteristic extension of an ‘arm’ like feature is seen in mature gametocytes of *H. leiothrichus* n. sp. and *H. homoleiothrichus* n. sp. ( Figure 2D–G, I, Figure 3D, G) along with no significant difference between the size, shape and number of pigment granules. It is to be noted that both *H. leiothrichus* n. sp. and *H. homoleiothrichus* n. sp. vary in their prevalence; *H. leiothrichus* n. sp. with a prevalence of 11.25% whereas *H. homoleiothrichus* n. sp. has a prevalence of 1.73%. We can speculate that this difference in the overall prevalence between the two parasites can be attributed to factors such as virulence of the parasite species, and availability of suitable vectors, or competition between lineages which warrants further investigation.

**Figure 5.**
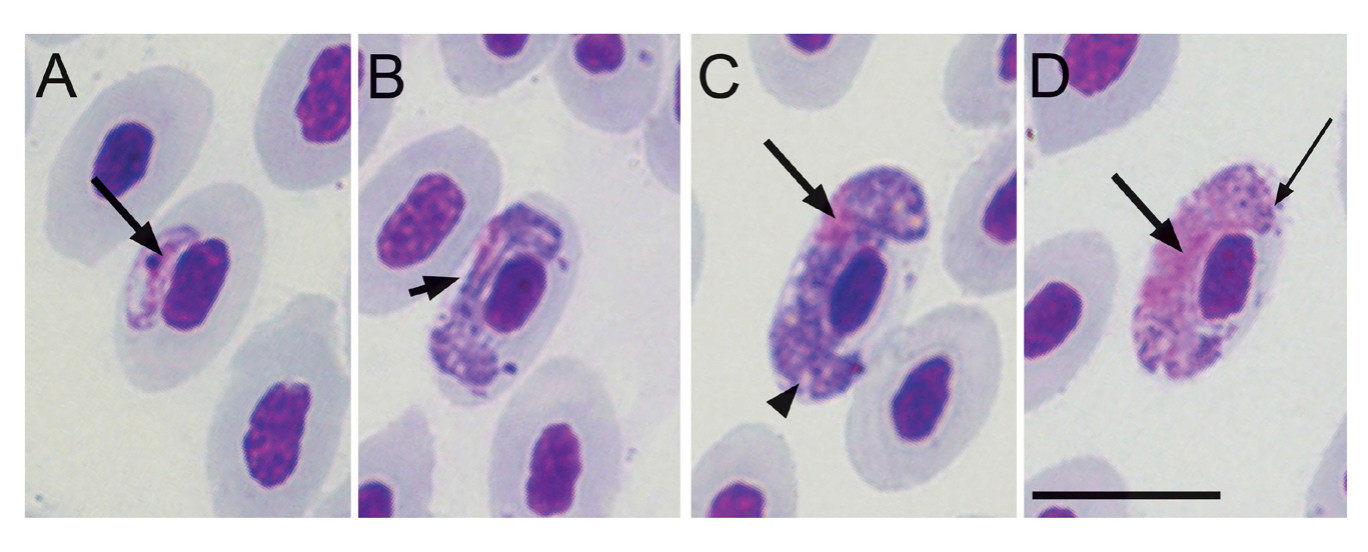
Gametocytes of *Haemoproteus timalus* (blood of the *Garrulax mitratus*; accession numbers 4062, in the collection of the International Reference Centre for Avian Haematozoa at the Queensland Museum, Australia); 5. ( **A**) growing gametocyte, ( **B**, **C**) macrogametocytes and ( **D**) microgametocyte. Scale bar = 10 µm. Long arrow points to the nucleus of the parasite; short arrow - indicates unfilled spaces between gametocytes and envelop of infected erythrocyte; arrow head shows pigment granules; long narrow arrow shows volutin granules.

*Haemoproteus leiothrichus* n. sp. and *H. homoleiothrichus* n. sp. are morphologically similar to *Haemoproteus timalus*, which was described from *Turdoides rubiginosus* in South Horr, Kenya ( Valkiūnas, 2005). There are the following key features that distinguish *H. timalus* and the 2 parasites described here: Dumbbell-like appearance is present in mature macrogametocytes and microgametocytes of *H. leiothrichus* n. sp. ( Figure 2G, H) and *H. homoleiothrichus* n. sp. ( Figure 3C, D) while in *H. timalus,* mature gametocytes lose this shape and are closely appressed to the nucleus and envelope of infected erythrocyte ( Figure 5C). Gametocytes of *H. leiothrichus* n. sp. and *H. homoleiothrichus* n. sp. more often do not entirely fill the poles of the erythrocytes ( Figure 2D, E, G, H, Figure 3D). Maximum NDR observed in *H. leiothrichus* n. sp. and *H. homoleiothrichus* n. sp. is more than that recorded in *H. timalus* ( Table 1). Two parasites described here have been observed to push the nucleus of the erythrocyte laterally so that it touches its host cell envelope; however, this feature is not seen in *H. timalus* ( Figure 5C, D). Fully grown gametocytes of *H. timalus* fill the poles of the erythrocyte ( Figure 5C) which is not always the case with *H. leiothrichus* n. sp. or *H. homoleiothrichus* n. sp.. The average number of pigment granules in *H. leiothrichus* n. sp. (n = 8.6) and *H. homoleiothrichus* n. sp. (n = 9.8) is lesser than that seen in *H. timalus* (n = 10.9).

## Discussion

We have described the morphology and provided molecular evidence for the 2 proposed avian haemoproteids *H. leiothrichus* n. sp. and *H. homoleiothrichus* n. sp. as new species. These are the first described species of parasites from the western and eastern Himalayan regions which also happen to be morphologically cryptic. Both parasite species appear to be restricted to laughingthrushes (family Leiothrichidae, formerly included in Timallidae). The chestnut-crowned laughingthrush *T. erythrocephalum* is a south Asian passerine, found in India, Bhutan, China and Nepal, and is one of the most common laughingthrushes seen in the Himalaya ( Rasmussen & Anderton, 2005). Previously, *T. erythrocephalum* has been described as an additional host for a morphologically described *Haemoproteus timalus* in the oriental region ( Bennett *et al.*, 1991). Recently, *H. timalus* has been recorded in a population of *Garrulax* (now *Trochalopteron*) *erythrocephalum* in south-east Asia ( Paperna *et al.*, 2008). Bennett & Campbell (1972) identified *Haemoproteus fallisi* in 2 timaliines, *Garrulax erythrocephalus* and *Leiothrix argentauris.* Subsequently, these identifications were considered in error and the parasites involved were referred to as *H. timalus* ( Bennett *et al.*, 1991). Bennett *et al.* (1991) described haemoproteids found in 43 species of timaliine birds as *H. timalus*, a hypothesis proposed to be tested ( Valkiūnas, 2005). However, this ambiguity in the identification of the parasite *H. timalus* has been further confounded by the lack of genetic information. Therefore, linking morphological studies with molecular phylogeny is crucial to understand parasite species divergence within a host species, or phylogenetically closely related host species.

Despite similar morphology with *H. timalus*, our described parasites contain several features that are absent in *H. timalus* which are important for taxonomical identification. These are, the presence of some dumbbell-like shape mature gametocytes, ‘arm’ like extensions of gametocytes, and lateral displacement of some nuclei of infected erythrocytes. By comparing *H. leiothrichus* n. sp. ( Figure 2) and *H. homoleiothrichus* n. sp. ( Figure 3) with the type material of *H. timalus* ( Figure 5), we can conclusively state the above mentioned differences. The lack of genetic sequence for *H. timalus* and its morphological similarity with *H. vireonis, H. coatneyi* and *H. passeris* as described by Valkiūnas (2005), makes it difficult to understand the genetic divergences among the parasites found in the same host family. The genetic divergence based on partial mitochondrial cytochrome *b* gene between *H. vireonis*, *H. coatneyi,* and *H. passeris* and *H. leiothrichus* n. sp. is 3.2%, 2.8% and 3.2% respectively and 4%, 3.6% and 3.2% respectively with *H. homoleiothrichus* n. sp. *H. leiothrichus* n. sp. is most closely related to *Haemoproteus homobelopolskyi* and *Haemoproteus magnus* with a genetic distance of 1.7%. Even though *H. homobelopolskyi* and *H. magnus* differ from *H. leiothrichus* n. sp. by a genetic distance as low as 1.7%, key morphological features describing *H. homobelopolskyi* and *H. magnus* is completely absent in *H. leiothrichus* n. sp. The most prominent feature of *H. magnus,* where it completely encircles the host erythrocyte and the presence of rod like pigment granules, are absent in *H. leiothrichus* n. sp. *Haemoproteus leiothrichus* n. sp. also significantly differs from *H. homobelopolskyi,* as the former is not seen completely enclosing the host nucleus or encircling it. Similarly *H. homoleiothrichus* n. sp. differs from *Haemoproteus tartakovskyi* with a genetic distance of 2.1%, but a significant morphological difference between these two parasites is observed despite the small genetic difference. *Haemoproteus tartakovskyi* enucleates the host cell and infected erythrocytes are hypertrophied, but these features are not observed in *H. homoleiothrichus* n. sp.

We described *H. leiothrichus* n. sp. and *H. homoleiothrichus* n. sp. as cryptic species, which share the same morphological features; however the phylogenetic position, a genetic divergence of 4.4%, and observed divergences with other *Haemoproteus* lineage recovered in resident Himalayan birds (F. Ishtiaq, unpubl. data) also provide evidence to separate the 2 species despite morphological similarities ( Figure 4).

*Haemoproteus* lineages differing by >5% are typically morphologically distinct ( Hellgren *et al.*, 2007), but there are some morphological species that have a divergence of less than 0.7% ( Outlaw & Ricklefs, 2014). Given the overlap in range and distribution of 2 *Haemoproteus* species described in laughingthrushes, a genetic distance of 4.4% suggests that these parasites are biologically independent entities and not a species complex diversified within the same host species.

In our phylogenetic analysis, *H. leiothrichus* n. sp. shows a divergence of 1.7 – 10.6% whereas *H. homoleiothrichus* n. sp. shows a divergence of 2.1 – 12.7% with other morphologically described *Haemoproteus* lineages associated with the subgenus *Parahaemoproteus*. Low genetic distances between reference *Haemoproteus* species and the 2 described species shows that minimal genetic variation can result in significant morphological variation.

Nilsson *et al.* (2016) showed that 5 morphologically cryptic species nested within a clade forming *H. majoris* were actually reproductively isolated entities and hence independent biological species. Based on partial mitochondrial cyt *b* sequences, the genetic divergence within the *H. majoris* clade ranged from 0.2 – 1.3% which is far lesser than the divergence recorded in our study on laughingthrushes.

Our study is the first ever survey of avian haemosporidians using both traditional microscopy and molecular methods conducted in the western and eastern Himalaya in India. This study shows that *H. leiothrichus* n. sp. is a widespread haemoproteid, infecting *T. erythrocephalum* and *T. variegatum* both in western and eastern Himalaya, while *H. homoleiothrichus* n. sp. retrieved in *T. erythrocephalum* and *T. lineatum* appears to be confined to western Himalaya. Given that all sampled species of laughingthrushes occur in sympatry, the described *Haemoproteus* species could be host-specific lineages harbored exclusively by laughingthrushes. The host ranges of *Haemoproteus* species are often restricted to a limited number of closely related host species ( Atkinson & van Riper, 1991; Fallon *et al.*, 2004; Pérez-Tris *et al.*, 2007; Savage & Greiner, 2004). During our large scale bird sampling in the Himalayan region, we have not found *H. leiothrichus* n. sp. and *H. homoleiothrichus* n. sp. infecting any other avian host. From that broader phylogenetic context, both species seem to be fixed on laughingthrushes, sharing the same morphology and phylogenetically distantly related. Extensive research on vector species would provide insights to host-specificity of these prevalent parasites.

## Data availability

The data underlying this study is available from Open Science Framework. Dataset 1: Laughingthrush parasites. https://doi.org/10.17605/OSF.IO/9GJ6Y ( Ishtiaq, 2018)

This dataset is available under a CC0 1.0 Universal

Mitochondrial cyt *b* lineage sequences for the two identified species have been deposited to GenBank: Accession numbers KY623720 and KY623721

## Supplementary material

Supplementary File 1: document containing the following supplementary table:

Table S1: Sequence divergence between eight morphologically described *Haemoproteus* sequences and *H. leiothrichus* n. sp. and *H. homoleiothrichus* n. sp. as analysed using the Kimura 2-parameter model. The rate variation among sites was modelled with a gamma distribution.

## References

[ref-1] Atkinson CT Van Riper C III: Pathogenicity and epizootiology of avian haematozoa: *Plasmodium, Haemoproteus,* and *Leucocytozoon* . In *Bird-parasite interactions: Ecology, evolution, and behavior.*Oxford University Press, London, U.K.,1991;19–48. Reference Source

[ref-3] Beadell JS Fleischer RC: A restriction enzyme-based assay to distinguish between avian hemosporidians. *J Parasitol.* 2005;91(3):683–685. 10.1645/GE-3412RN 16108566

[ref-2] Beadell JS Gering E Austin J: Prevalence and differential host-specificity of two avian blood parasite genera in the Australo-Papuan region. *Mol Ecol.* 2004;13(12):3829–3844. 10.1111/j.1365-294X.2004.02363.x 15548295

[ref-4] Bennett GF Campbell AG: Avian haemoproteidae. I. Description of *Haemoproteus fallisi* n. sp. and a review of the haemoproteids of the family Turdidae. *Can J Zool.* 1972;50(10):1269–1275. 10.1139/z72-172 4628849

[ref-5] Bennett GF Bishop MA Peirce MA: The species and distribution of the haemoproteids of the avian family Muscicapidae *sensu latu* (Passeriformes). *J Nat Hist.* 1991;25(1):23–43. 10.1080/00222939100770041

[ref-6] Bensch S Hellgren O Pérez-Tris J: MalAvi: a public database of malaria parasites and related haemosporidians in avian hosts based on mitochondrial cytochrome *b* lineages. *Mol Ecol Resour.* 2009;9(5):1353–1358. 10.1111/j.1755-0998.2009.02692.x 21564906

[ref-7] Clark NJ Clegg SM Lima MR: A review of global diversity in avian haemosporidians ( *Plasmodium* and *Haemoproteus*: Haemosporida): new insights from molecular data. *Int J Parasitol.* 2014;44(5):329–338. 10.1016/j.ijpara.2014.01.004 24556563

[ref-8] Drummond AJ Rambaut A: BEAST: Bayesian evolutionary analysis by sampling trees. *BMC Evol Biol.* 2007;7:214. 10.1186/1471-2148-7-214 17996036 PMC2247476

[ref-9] Fallon SM Ricklefs RE Latta SC: Temporal stability of insular avian malarial parasite communities. *Proc Biol Sci.* 2004;271(1538):493–500. 10.1098/rspb.2003.2621 15129959 PMC1691613

[ref-10] Feldman RA Freed LA Cann RL: A PCR test for avian malaria in Hawaiian birds. *Mol Ecol.* 1995;4(6):663–673. 10.1111/j.1365-294X.1995.tb00267.x 8564006

[ref-11] Godfrey RD Jr Fedynich AM Pence DB: Quantification of hematozoa in blood smears. *J Wildl Dis.* 1987;23(4):558–565. 10.7589/0090-3558-23.4.558 3119870

[ref-12] Hellgren O Waldenström J Bensch S: A new PCR assay for simultaneous studies of *Leucocytozoon, Plasmodium,* and *Haemoproteus* from avian blood. *J Parasitol.* 2004;90(4):797–802. 10.1645/GE-184R1 15357072

[ref-35] Hellgren O Waldenström J Peréz-Tris J: Detecting shifts of transmission areas in avian blood parasites: a phylogenetic approach. *Mol Ecol.* 2007;16(6):1281–1290. 10.1111/j.1365-294X.2007.03227.x 17391413

[ref-13] Ishtiaq F: Laughingthrush parasites.2018 10.17605/OSF.IO/9GJ6Y

[ref-14] Ishtiaq F Gering E Rappole JH: Prevalence and diversity of avian hematozoan parasites in Asia: a regional survey. *J Wildl Dis.* 2007;43(3):382–398. 10.7589/0090-3558-43.3.382 17699077

[ref-15] Nandi NC Bennett GF: The prevalence distribution and checklist of avian haematozoa in the Indian subcontinent. *Rec Zool Surv India.* 1997;96(1–4):83–150. Reference Source

[ref-16] Nilsson E Taubert H Hellgren O: Multiple cryptic species of sympatric generalists within the avian blood parasite *Haemoproteus majoris*. *J Evol Biol.* 2016;29(9):1812–1826. 10.1111/jeb.12911 27262030

[ref-17] Outlaw DC Ricklefs RE: Species limits in avian malaria parasites (Haemosporida): how to move forward in the molecular era. *Parasitology.* 2014;141(10):1223–1232. 10.1017/S0031182014000560 24813385

[ref-18] Palinauskas V Žiegytė R Ilgūnas M: Description of the first cryptic avian malaria parasite, *Plasmodium homocircumflexum* n. sp., with experimental data on its virulence and development in avian hosts and mosquitoes. *Int J Parasitol.* 2015;45(1):51–62. 10.1016/j.ijpara.2014.08.012 25449950

[ref-19] Paperna I Keong MSC May CYA: Haemosporozoan parasites found in birds in Peninsular Malaysia, Singapore, Sarawak and Java. *Raffles Bull Zool.* 2008;56(2):211–243. Reference Source

[ref-20] Pérez-Tris J Hellgren O Krizanauskiene A: Within-host speciation of malaria parasites. *PLoS One.* 2007;2(2):e235. 10.1371/journal.pone.0000235 17311104 PMC1794596

[ref-21] Perkins SL: Species concepts and malaria parasites: detecting a cryptic species of *Plasmodium*. *Proc Biol Sci.* 2000;267(1459):2345–2350. 10.1098/rspb.2000.1290 11413654 PMC1690816

[ref-22] Schneider CA Rasband WS Eliceiri KW: NIH Image to ImageJ: 25 years of image analysis. *Nat Methods.* 2012;9(7):671–675. 10.1038/nmeth.2089 22930834 PMC5554542

[ref-23] Rasmussen PC Anderton JC: Birds of south Asia: the Ripley guide. Vols. 1 and 2. Smithsonian Institution, Washington, D.C., and Lynx Edicons, Barcelona, Spain, 378 p. and 683 p., respectively.2005.

[ref-24] Sambrook J Fritsch EF Maniatis T: Molecular cloning- A laboratory manual. Cold Spring Harbor Laboratory Press, Cold Spring Harbor, New York,1989;1626 Reference Source

[ref-25] Savage AF Greiner EC: Hematozoa of the avian family Brachypteraciidae (the ground-rollers). *J Parasitol.* 2004;90(6):1468–1472. 10.1645/GE-227R 15715245

[ref-26] Sehgal RN Hull AC Anderson NL: Evidence for cryptic speciation of *Leucocytozoon* spp. (Haemosporida, Leucocytozoidae) in diurnal raptors. *J Parasitol.* 2006;92(2):375–379. 10.1645/GE-656R.1 16729697

[ref-27] Tamura K Peterson D Peterson N: MEGA5: molecular evolutionary genetics analysis using maximum likelihood, evolutionary distance, and maximum parsimony methods. *Mol Biol Evol.* 2011;28(10):2731–2739. 10.1093/molbev/msr121 21546353 PMC3203626

[ref-28] Valkiūnas G: Avian malaria parasites and other haemosporidia.CRC Press, Boca Raton, Florida,2005;946 Reference Source

[ref-29] Valkiūnas G Iezhova TA Loiseau C: New species of haemosporidian parasites (Haemosporida) from African rainforest birds, with remarks on their classification. *Parasitol Res.* 2008;103(5):1213–1228. 10.1007/s00436-008-1118-x 18668264

