# Supplementary material for: Molecular characterization and morphological description of cryptic haemoproteids in the laughingthrushes (Leiothrichidae) in the western and eastern Himalaya, India
Source: Wellcome Open Res. 2018 Aug 1;3:94. [Version 1] doi: 10.12688/wellcomeopenres.14675.1 (PMC6221074; doi:10.12688/wellcomeopenres.14675.1)
Supplement: Supplementary file 1 [file wellcomeopenres-3-15977-s0000.tgz › 85046917-b157-488a-98a2-017f4eb0276f.docx]

## Electronic supplementary material:

**Molecular characterization and morphological description of cryptic Haemoproteids in the laughingthrushes** (**Leiothrichidae) in the western and eastern Himalaya, India**

**Farah Ishtiaq**^1*^**, Megha Rao**^1^ **and Vaidas Palinauskas** ^2*^

Farah Ishtiaq^1^, Centre for Ecological Sciences, Indian Institute of Science, Bangalore-560012, India

*Email: ishtiaq.farah@gmail.com

Megha Rao, Centre for Ecological Sciences, Indian Institute of Science, Bangalore-560012, India

Vaidas Palinauskas^2^ , Senior Researcher, P.B. Šivickis’ Laboratory of Parasitology, Institute of Ecology, Nature Research Centre, Akademijos 2, Vilnius 2100, LT-08412, Lithuania

*Email: vaidas@ekoi.lt

*joint corresponding authors

Table S1: Sequence divergence between eight morphologically described *Haemoproteus* sequences and *H. leiothrichus* n. sp. and *H. homoleiothrichus* n. sp. as analysed using the Kimura 2-parameter model. The rate variation among sites was modelled with a gamma distribution.

| Lineage | TROERY02 TROERY01 PLOMEL01 PADOM05 | | | | | | |
| --- | --- | --- | --- | --- | --- | --- | --- |
| TROERY02_*H_homoleiothrichus*_KY623721 |  |  |  |  |  |  |  |
| TROERY01_*H_leiothrichus*_KY623720 | 0.044 |  |  |  |  |  |  |
| PLOMEL01_*H_homobelopolskyi*_HQ386240 | 0.032 | 0.017 |  |  |  |  |  |
| PADOM05_*H_passeris*_HM146898 | 0.032 | 0.032 | 0.014 |  |  |  |  |
| ANSOM01_*H_coatneyi*_KM211350 | 0.036 | 0.028 | 0.052 | 0.010 |  |  |  |
| ROFI1_*H_magnus*_DQ060769 | 0.025 | 0.017 | 0.014 | 0.014 |  |  |  |
| ALARV01_*H_tartakovskyi*_GU289671 | 0.021 | 0.021 | 0.010 | 0.017 | 0.010 |  |  |
| VIGIL01_*H_vireonis*_FJ168561 | 0.040 | 0.032 | 0.021 | 0.024 | 0.021 |  |  |
| CCF5_*H_majoris*_DQ060765 | 0.044 | 0.028 | 0.024 | 0.028 | 0.017 | 0.003 |  |
| CB1_*L_majoris*_AY393804 | 0.253 | 0.225 | 0.225 | 0.238 | 0.211 | 0.224 | 0.217 |
