# Supplementary material for: Molecular characterization and morphological description of cryptic haemoproteids in the laughingthrushes (Leiothrichidae) in the western and eastern Himalaya, India
Source: Wellcome Open Res. 2018 Aug 1;3:94. [Version 1] doi: 10.12688/wellcomeopenres.14675.1 (PMC6221074; doi:10.12688/wellcomeopenres.14675.1)
Supplement: Peer review history

# Peer review: Referee response for version 1

**DOI:** 10.21956/wellcomeopenres.15977.r34119

**Published:** 2018-11-14

**Authors:** Pavel Munclinger

**Affiliations:** 1 Department of Zoology, Faculty of Science, Charles University, Prague, Czech Republic

The manuscript describes two new parasite cryptic species from interesting region which is far less studied than the American or Euro-African systems. The host species belongs to typical Asian bird family which is understudied for avian malaria. Hence I have found this contribution very welcome and highly needed. The descriptions are careful, accurate and appropriately based on morphology as well as molecular methods (cytochrome b sequencing). Microscopic slides on photos are of excellent quality and the manuscript is very well written.

 I have just few minor comments:

 The restriction enzyme based assay detects also Leucocytozoon. However, no Leucocytozoon infection is mentioned in the manuscript. Does it mean that the Leucocytozoon prevalence was zero?

 I think the initial PCR primers should be also mentioned for the nested PCR detection method.

 Two sequences were deposited into the Genebank, but it seems to me from the Figure 4 that there has been some intra-species sequence variation in Haemoproteus homoleiothrichus sp. nov. (TROERY02). I miss any comment on intra-species sequence differences in the manuscript. Please add some info.

 I have found this sentence rather awkward: “…the most appropriate substitution model (GTR+G) according to the Akaike Information Criterion was implemented MEGA version 5.2…” I think the authors used a model test based on AIC which is implemented in MEGA.

 The Kimura 2-parameter evolution model is frequently used to calculate distances. However, I wonder how much this model fits given the fact that GTR+G was suggested as the best model for the given alignment. Can the authors discuss it somehow?

 I have no idea why and how 103 out of 231 samples with blood smears were chosen for analysis. Have the 103 samples been selected just randomly? Perhaps it would be too time consuming to look at all slides but in such a case why the PCR positive samples have not been preferentially selected?

 No Leucocytozoon on slides?

 How much the PCR and microscopy detection agreed?

 The localities are very distant. Was there any difference in prevalence between the localities?

 There should be Bayesian phylogeny on Figure 4 (Is it really Bayesian one?). It is rather strange that the support is given as bootstrap values. Posterior probability is regularly used on Bayesian trees instead.

 The authors invested a lot of time into microscopy examination. Hence they may consider mentioning also parasitemia?

I have read this submission. I believe that I have an appropriate level of expertise to confirm that it is of an acceptable scientific standard.

# Peer review: Referee response for version 1

**DOI:** 10.21956/wellcomeopenres.15977.r34120

**Published:** 2018-11-02

**Authors:** Yukita Sato

**Affiliations:** 1 Graduate School of Veterinary Medicine, Nihon University, Fujisawa, Japan

This article describes newly recognized two *Haemoproteus* species from laughingthrusehs in India with robust data based on both morphology and molecular biology. Authors showed detailed characteristic features of *H*. *leiothrichus *n. sp. and *H. homoleiothrichus *n. sp. by clear figures and phylogenetic trees with clear distinguish points against *H. timalus*. I recommend this article for other researchers for their information and understanding of present status of Haemoproteus distribution among laughingthrushes. I agree with former reviewr's comments for clear descriptions in some parts of the manuscript.

I have read this submission. I believe that I have an appropriate level of expertise to confirm that it is of an acceptable scientific standard.

# Peer review: Referee response for version 1

**DOI:** 10.21956/wellcomeopenres.15977.r33615

**Published:** 2018-08-15

**Authors:** Peter Shurulinkov

**Affiliations:** 1 National Museum of Natural History –Sofia, Bulgarian Academy of Sciences, Sofia, Bulgaria

The article presents valuable findings with description of two new cryptic haemoproteid species in a poorly studied group of Indian passerine birds. The descriptions are  proved very well with measurements, photographs and text. A taxonomic remarks were also discussed and the position of the new species in a genetic aspect is commented. It is important to note that the study makes connection between genetic findings of the haemoproteid lineages and their morphospecies studied by microscopy.  I think the data is well presented in a good scientific style. I have the following questions/remarks: 

- It is not mentioned in material and methods why exactly 103 out of 231 samples were screened microscopically.
- On Figure 2 and Figure 3 it is not clear what are the objects pointed to by the arrows.
- The text explaining the morphological differences between the two newly described haemoproteid species is repeated in the section Results (Remarks) and in the section Discussion. It should be avoided and commented at one of the places (where the authors decide).

I have read this submission. I believe that I have an appropriate level of expertise to confirm that it is of an acceptable scientific standard.
